# Spatial ecology of the Vicuña (*Lama vicugna*) in a high Andean protected area

**DOI:** 10.1093/jmammal/gyad018

**Published:** 2023-03-16

**Authors:** Harshad Karandikar, Emiliano Donadio, Justine A Smith, Owen R Bidder, Arthur D Middleton

**Affiliations:** Department of Environmental Science, Policy & Management, University of California, Berkeley, California 94720, USA; Rewilding Argentina, Estancia La Ascensión, Los Antiguos, Santa Cruz 9041, Argentina; Department of Wildlife, Fish, Conservation Biology, University of California, Davis, California 95616, USA; Department of Environmental Science, Policy & Management, University of California, Berkeley, California 94720, USA; Department of Environmental Science, Policy & Management, University of California, Berkeley, California 94720, USA

**Keywords:** a-LoCoH, camelid, diel migrations, home range, *Lama vicugna*, territoriality, vicuña

## Abstract

The study of animal space use is fundamental to effective conservation and management of wildlife populations and habitats in a rapidly changing world, yet many species remain poorly described. Such is the case for the spatial ecology of the Vicuña–a medium-sized wild camelid that plays a critical role, both as a consumer and as prey, in the high Andean food web. We studied patterns of space use of 24 adult female vicuñas from April 2014 to February 2017 at the southern edge of its range. Vicuñas showed strong fidelity to their home range locations across the study period and shared large portions of their home ranges with vicuñas from other family groups. Vicuña home ranges in our study were considerably larger than previous estimates across the range of the species. Variation in environmental and terrain factors and the associated risk of predation affected vicuña diel migration distance but not home range size or overlap. Our study offers new ecological insights into vicuña space use that can inform conservation and management efforts of vicuñas and other social ungulates.

## Abstract (es)

El estudio del uso del espacio en animales es fundamental para la conservación y gestión eficaz de sus poblaciones y hábitats silvestres en un mundo que cambia rápidamente, sin embargo muchas especies siguen estando mal descritas. Tal es el caso del estudio de ecología espacial de la vicuña, un camélido silvestre de tamaño mediano que tiene un papel crítico en la red trófica altoandina. Estudiamos el uso del espacio de 24 hembras adultas de vicuñas desde abril de 2014 hasta febrero de 2017 en el extremo sur del área de distribución de la especie. Las vicuñas mostraron una gran fidelidad en la ubicación de sus áreas de acción durante el período de estudio y compartieron gran parte de sus áreas de acción con otros grupos familiars. Las áreas de acción de las vicuñas en nuestro estudio fueron considerablemente más grandes que las estimaciones previas en todo el rango de la especie. Variacíon en factores ambientales y topográficos, y el riesgo asociado de depredación afectaron las distancias de las migraciones diarias de las vicuñas, pero no afectaron el tamaño del área de acción. Nuestro estudio reporta nuevos datos ecológicos sobre el uso del espacio de vicuñas que pueden informar los esfuerzos de conservación y manejo de esta especie y otros ungulados sociales.

Conserving ungulates and their ecological roles requires comprehensive understanding of their behavior, natural history, and space use–however, many species have not been adequately described to facilitate targeted conservation approaches. Within ungulates, considerable diversity exists in space use, including nomadic behavior without site fidelity, home ranges without territoriality, lekking, year-round territoriality, and seasonal territoriality (Lott 1991)–driven by factors including behavioral and genetic plasticity and a multitude of environmental and ecological variables affecting space use (Maher and Lott 2000). Such variation in space use and social structure signifies the wide range of habitat requirements across species. Understanding animal space use, especially the common yet complex phenomena of home ranging behavior and territoriality (Owen-Smith 1977), is fundamental to effective conservation and management of wildlife populations and habitats in a rapidly changing world.

The Vicuña (*Lama vicugna*) is a medium-sized wild camelid endemic to the high Andes of South America (Koford 1957; Franklin 1974) that plays a critical role in the high Andean food web and is an important food item for carnivores (Donadio et al. 2010; Donadio and Buskirk 2016) and scavengers (Perrig et al. 2017). The Vicuña is the most abundant large herbivore in the region and has important effects on the plant community (Donadio and Buskirk 2016). Indiscriminate hunting in the 19th and 20th centuries led to a precipitous decline in vicuña populations across their range, before the species received legal protection under the Convention for the Conservation and Management of the Vicuña in 1979 and recovered in many areas during the late 20th century (McNeill et al. 2009). While the northern subspecies of the Vicuña, *L. v. mensalis*, is no longer in danger of extinction, the southern subspecies, *L. v. vicugna*, is still threatened (Bonacic and Gimpel 2003; Acebes et al. 2018). Many wild vicuña populations continue to be highly managed or are otherwise impacted by human use of the landscape (McLaren et al. 2018). In some areas, vicuñas are periodically captured and sheared for their highly valued fiber, which can alter social behavior (Bonacic and Galaz 2001; but see Arzamendia et al. 2018) and increase stress levels (Bonacic and Macdonald 2003). Attempts have even been made to hybridize vicuñas and alpacas (*L. pacos*) to improve fiber quality and production (Lichtenstein 2009). More recently, outbreaks of mange, a highly contagious disease caused by mites (*Sarcoptes scabiei*), have heavily impacted some populations (Monk et al. 2022).

Previous studies on vicuña space use and social behavior, based on visual observation of known individuals, have suggested that family group territories are exclusive and well-defended, with high site fidelity (Koford 1957; Franklin 1974; Bosch and Svendsen 1987; Arzamendia et al. 2018). Other studies, however, contend that vicuña families tend to tolerate some territory overlap (Vilá 1994) and that territoriality is not universal in the species (Vilá and Roig 1992; Cassini et al. 2009). Vicuñas are usually sedentary (i.e., do not undertake seasonal migrations) and tend to only use small portions of available suitable habitat (Cassini et al. 2009). Increased mobility in some vicuña populations has been ascribed to human disturbance (Vilá 2000). About 60% of vicuñas live in Permanent Territorial Family Groups (Franklin 1974, 1976) that generally comprise one male, three to four females, and one to two offspring (Cassini 2009). Vicuña families have also been reported to maintain distinct feeding (day) and sleeping (night) territories (Franklin 1974), although other studies suggest that this behavior may not be universal (Koford 1957; Menard 1982). While data obtained through visual observations offer critical information about behavior that is impossible to determine using remotely sensed locational information (e.g., definitive evidence about territory defense and thus territoriality), advances in biologging technologies now allow for more fine-scale, continuous, and comprehensive analysis of animal space use (Kays et al. 2015; Wilmers et al. 2015) compared to the limited number of observations possible through visual methods.

We aim to investigate space use in a population of wild, unmanaged vicuñas using the first available GPS location data set for the species and compare this with the current understanding of vicuña spatial ecology. The main objectives of this work are to: (1) offer the first estimates of vicuña home range sizes using GPS locations and understand the relationship between forage availability, family group size, and home range size; and (2) assess the impact of environmental factors on home range size, overlap, and diel migrations. Environmental conditions, including resource availability and distribution, may affect ungulate space use and space sharing. The habitat productivity hypothesis, for example, suggests that ungulate home ranges tend to be smaller in areas with higher productivity (Harestad and Bunnel 1979; Seigle-Ferrand et al. 2021), whereas the resource dispersion hypothesis supports home range sharing when forage availability is limited and highly clumped (Johnson et al. 2002). Environmental conditions may also impact daily movements, with diel migrations previously reported in the system (Smith et al. 2019a). Vicuñas in our study system used two distinct, mutually exclusive areas, offering a unique opportunity to contrast behavioral and space use differences associated with varying environmental conditions within the same broader landscape. At the study system level, environmental conditions differ considerably from other areas in the vicuña range, offering an opportunity to understand vicuña space use and examine the differences in space use and behavior in differing environments.

First, we estimate vicuña home range sizes and test the relationships between range size and environmental factors and family size. We then investigate differences in range sizes across seasons (growing and nongrowing) and sites. We hypothesize that differences in vegetation and terrain–including forage availability and distribution, elevation, and slope–lead to differences in space use. We predict that vicuña home ranges will be smaller in the site with higher forage availability and during the growing period. Next, we investigate space sharing between vicuñas. We hypothesize that environmental conditions affect space sharing and predict that: (a) vicuña ranges will generally overlap due to the limited availability and clumped distribution of forage; and (b) range overlap will decrease in the growing period due to increased forage availability. Finally, we investigate vicuña diel migrations between day and night ranges. We hypothesize that vicuñas adjust their daily movements in response to environmental conditions. We predict that: (a) vicuñas in the site with less heterogeneity will move longer daily distances; and (b) vicuñas will move longer daily distances in the nongrowing periods due to reduced availability of forage.

## Materials and Methods

### Study area and species.—

The study was conducted in San Guillermo National Park, San Juan Province, Argentina, between April 2014 and February 2017. The park is at the southern edge of the vicuña range and is located in a remote part of the central Andes mountains (29°14ʹS, 69°21ʹW), with limited access to visitors and consequently very low levels of human disturbance (Donadio and Buskirk 2006). The park is in a semiarid region at an altitude of 2,000–5,600 m, with rainfall largely limited to a period from January to March, leading to a narrow growing season in mid and late summer (Salvioli 2007; Donadio et al. 2012). Three main habitat types characterize the park: medium-altitude plains, steep canyons, and meadows. The plains and canyons comprise a total of 96% of the park area, whereas the meadows that exist in patches in the plains or near drainage features comprise of 4% of the area (Donadio and Buskirk 2016). Meadows contain fertile soils and high moisture levels with species such as *Juncus* spp., *Carex* spp., *Scirpus* spp., and *Festuca* spp.–whereas the other areas are dominated by perennial *Jarava* spp. and *Stipa* spp. grasses (Donadio and Buskirk 2016). Population densities in the park at the time of our study were estimated at 9.5–12.7 vicuñas/km^2^ (Donadio et al. 2012). Guanacos (*L*. *guanicoe*) are considerably less abundant in the landscape, occurring at densities of 1 guanaco/km^2^ (Puig and Videla 2007).

We deployed GPS collars (GPS 6000SD, Lotek) on 24 adult female vicuñas under permit #DCM 455 and subsequent renewals issued by the Administración de Parques Nacionales, Argentina. Prior to collaring, vicuñas were observed to identify animals from distinct family groups. Vicuña family groups were observed to be very cohesive and moved together when approached for darting. Vicuñas were darted from a truck or by approaching them slowly on foot, from distances ranging between 15–42 m. Carfentanil (0.03–0.06 mg/kg) with Naltrexone (100 mg Naltrexone/1 mg Carfentanil) and Thiafentanil oxalate (0.06–0.1 mg/kg) antagonized with Naltrexone (10 mg Naltrexone/1 mg Thiafentanil) were used. Established mammal handling guidelines (Sikes and Gannon 2011) were followed during animal capture and handling. The 24 vicuñas consisted of 13 and 11 females collared, respectively, in two sites within the park: (1) Llano de los Leones in the north; and (2) San Guillermo Canyon in the center of the park. Llano de los Leones comprises a large meadow with high forage availability within a large open plain with low productivity, with an elevation range of 3,360–4,031 m and low average slope angle. The San Guillermo Canyon, with an elevation range of 3,312–3,925 m, had higher forage availability but with a more heterogeneous distribution. San Guillermo Canyon also had higher heterogeneity in elevation and slope (Smith et al. 2019b). We conducted our analyses with a total of 95,872 location points from 24 individual vicuñas using a 3-h fix rate. Not all animals were monitored for the duration of the study period–the start and end dates of location data available for each vicuña are listed in Supplementary Data SD1.

### Vicuña group composition, size, and site fidelity.—

Group composition and size were documented during and after collaring of vicuñas. Although previous studies on the species indicate strong territorial behavior, we first conducted a site fidelity analysis for each vicuña to establish a quantitative basis for home range studies, using Mean Squared Distance and Linearity Index as metrics (Munger 1984). Site fidelity analysis compares differences between actual movements and multiple random walks (Spencer et al. 1990). We used the *reproducible home ranges* (rhr) package (Signer and Balkenhol 2015) in R for this initial analysis. We used the range shift test in the *marcher* package (Gurarie and Cheraghi 2017) to check for migratory behavior and range shifts. In cases where the range shift test could not offer conclusive evidence for the absence of a range shift, we calculated the migration distance and range shift index metrics (Gurarie et al. 2017) using the *marcher* package (Gurarie and Cheraghi 2017).

### Home range estimation.—

Vicuña home ranges were calculated separately across the study period, for different seasons and periods of the day (explained below). The adaptive local convex hull (a-LoCoH) method was primarily used for determining vicuña home ranges. The LoCoH method was favored over other home range estimators to calculate home range size because it more tightly outlines the areas utilized by the focal animal (Getz and Wilmers 2004), important from the perspective of understanding home range overlap. Of the three LoCoH approaches, we used the a-LoCoH method, as it is considered superior to the r and k methods (Getz et al. 2007). Optimal kernel parameter (*a*) values were determined for each vicuña by calculating home range areas for multiple values of *a* and selecting the value where the home range size–number of recorded locations curve tends to asymptote (Ryan et al. 2006; Fletcher and Fortin 2018). We used the heuristic value for *a* for some individuals where the optimal value could not be determined through the plots (Getz et al. 2007). In addition, we also calculated core ranges using 50% minimum convex polygon (MCP) and autocorrelated kernel density estimation (AKDE; Fleming et al. 2015) to enable better comparison with earlier studies on vicuñas.

### Overall and seasonal ranges.—

Vicuña home ranges were estimated in four ways: (1) 50% day ranges for the study period (henceforth referred to as overall core ranges), using day locations for the entire duration of the study; (2) 95% day ranges for each season (referred to as seasonal home ranges); (3) 50% day ranges for each season (referred to as seasonal core ranges); and (4) 50% night ranges for each season (referred to as night ranges). Overall core ranges were calculated to understand vicuña day use in the area over the duration of the study, whether this space use differed between the Llano de los Leones and the San Guillermo Canyon sites, and to evaluate possible movement between these two areas during the study period. Seasonal home ranges, seasonal core ranges, and night ranges were calculated for two periods in each year based on plant phenology–the nongrowing period from June to November, and a growing period from December to May (Donadio et al. 2012). For the day and night range estimation, the seasonal location data were separated into four categories according to the time of the day–dawn, day, dusk, and night–using the *sunriset* function in the maptools package in R (Bivand 2020). Since previous studies indicated daily movement occurred between day and night territories during dawn and dusk (Franklin 1974), we estimated seasonal core ranges and night ranges to identify important day and night areas after excluding points during dawn and dusk. Although rainfall in the park is largely limited to the months of January to March (Donadio et al. 2012), we included the months of December, April, and May in the growing period to ensure that home ranges were calculated for similar intervals and could be compared across these periods. Seasonal home ranges, seasonal core ranges, and night ranges were thus calculated for four distinct periods (nongrowing 2014, growing 2014, nongrowing 2015, and growing 2015) with data from 17, 13, 19, and 13 individuals, respectively. We did not conduct a seasonal analysis for the nongrowing 2016 and growing 2016 periods due to low sample sizes. The number of individuals varied across seasons due to multiple collaring phases and natural mortalities. We tested for the influence of resource availability on vicuña space use by calculating correlations between the seasonal core range size and mean Normalized Difference Vegetation Index (NDVI) and between seasonal core range size and family size. Mean NDVI values were calculated using Google Earth Engine (Gorelick et al. 2017) from LANDSAT-8 imagery for each of the identified seasonal periods. For analyzing differences in home range sizes for the same vicuñas across seasons, we used the Friedman test (Friedman 1937).

### Home range overlap.—

Seasonal core ranges were used for calculating overlap between vicuñas. The proportion of the seasonal core range of each vicuña individual that was shared with one or more other vicuñas from other families was calculated to determine exclusive-use areas and identify individuals that did not share home ranges. Next, we assessed whether overlap percentages changed across seasons for the same vicuñas, to understand if seasonal differences might be associated with patterns of range overlap. We used the Friedman test (Friedman 1937) to analyze differences in range overlap for the same vicuñas across seasons.

### Diel migrations—

To investigate the influence of resource availability and seasonality on diel migrations, we measured the distances moved by vicuñas between the centroids of the day and night areas on a daily basis. Wilcoxon ranked sum tests were used to analyze differences between daily distances moved in the two regions. Differences in daily distances moved between nongrowing and growing periods were analyzed using the Welch two-sample *t*-test.

## Results

### Vicuña family composition, group size, and site fidelity.—

**Fig. 1.**
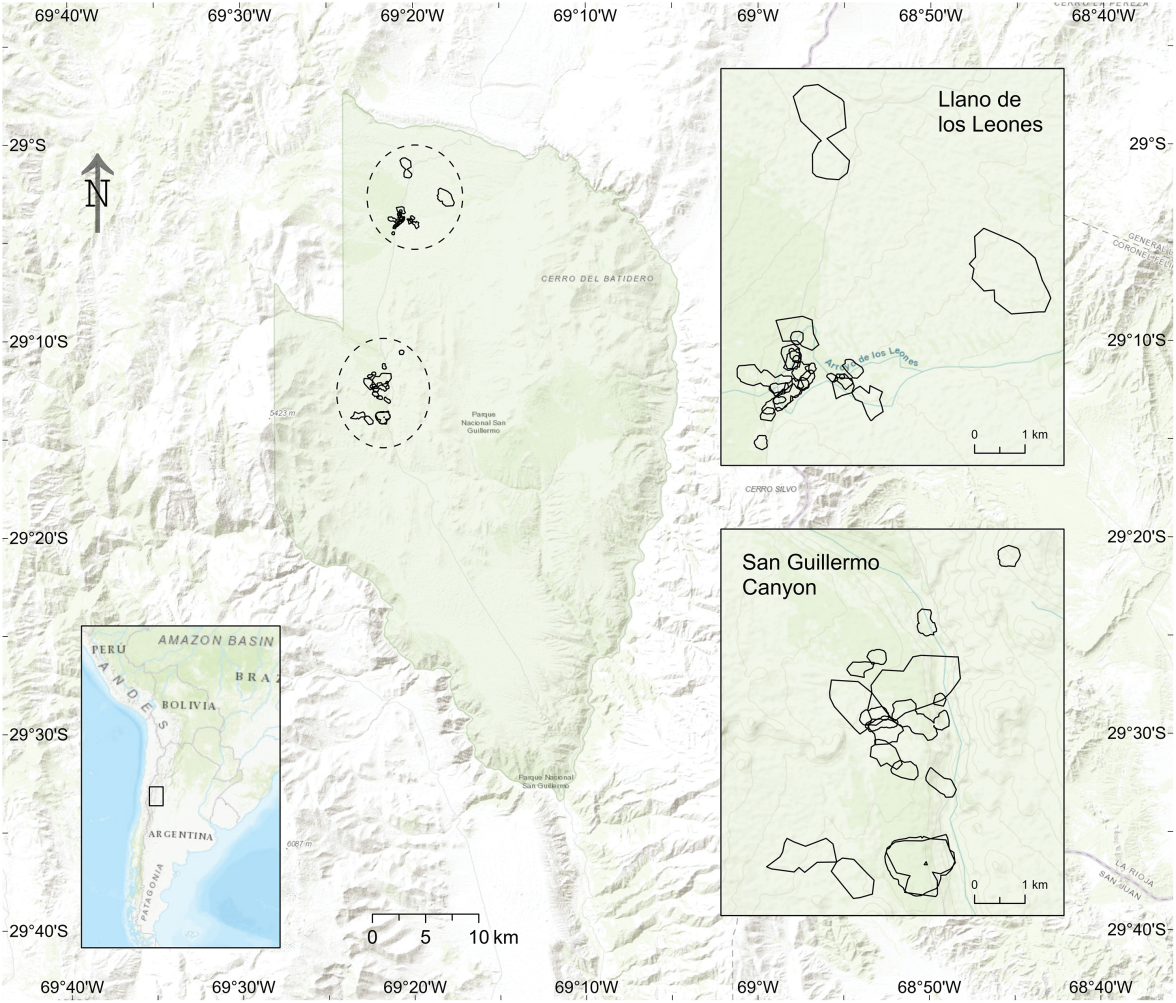
—Vicuña core ranges in the Llano de los Leones (north) and the San Guillermo Canyon (south) areas in San Guillermo National Park for the duration of the study. No vicuña was observed to move between the two sites during the study period.

Families with collared vicuñas included on average 3 (range 1–6) females and 2.1 (range 1–4) offspring. Visual inspection of plots generated by the *rhr* package offered evidence for site fidelity for all monitored individuals except one, where the result was inconclusive. The range shift test indicated no range shift for six vicuñas. For the remaining 18 vicuñas, although the range shift test was inconclusive, the largest ‘migration distance’ value of 0.043 km, with a range shift index value of 0.043, indicated that these vicuñas also did not shift ranges during the study. We did not find any movement between the two sites within the park (Fig. 1).

### Home range size and variation in sizes.—

The mean (± *SD*) overall core range sizes from our study were 0.53 (± 0.81) km^2^. Home range sizes using other methods and a comparison of home range sizes from previous studies are summarized in Table 1. Contrary to our predictions, we found little evidence for differences in range sizes between the Llano de los Leones and San Guillermo Canyon sites, regardless of type of home range examined (i.e., overall, seasonal, core; Supplementary Data SD2). One exception was for seasonal core ranges in the nongrowing period in 2014, where the average size of the seasonal core range for the San Guillermo Canyon (0.25 ± 0.10 km^2^, $\bar{x}$ ± *SD*) was significantly higher (*P* = 0.04) than for the Llano de los Leones (0.11 ± 0.14 km^2^, $\bar{x}$ ± *SD*). Seasonal core range sizes varied significantly across seasons (Friedman’s chi-squared = 8.35, d.f. = 3, *P* = 0.04)–however, the effect size was small (Kendall’s *W* = 0.253), and a post hoc Wilcoxon test with a Bonferroni correction resulted in no significant differences across pairs of seasons. Seasonal core range sizes did not vary significantly across seasons. We found no significant correlations between seasonal core range size and mean NDVI, except for the nongrowing 2015 period, when we found a significant but weak negative correlation. We also found weak, but not statistically significant, positive correlations between seasonal core range size and family size.

**Table 1.** —A comparison of 50% home range sizes for vicuñas for the duration of the study in San Guillermo National Park using adaptive local convex hull (a-LoCoH), autocorrelated kernel density estimation (AKDE), and minimum convex polygon (MCP) with vicuña home range sizes reported in previous studies.

Location	Method	Home range size (km^2^)	Reference
Huaylarco, Peru	MCP^a^	0.13^b^	Koford (1957)
Pampas Galeras Reserve, Peru	MCP^a^	0.18	Franklin (1976)
Pozuelos Biosphere Reserve, Argentina	KDE	0.19	Arzamendia et al. (2018)
San Guillermo National Park, Argentina	a-LoCoH	0.53	—
	AKDE	0.35	—
	MCP	2.42	—

^a^Equivalent to an MCP; however, home ranges were delineated manually using visual observations.

^b^Median value reported. All other values are mean.

### Range overlap.—

Vicuñas in San Guillermo National Park shared large portions (38.1 ± 37.38%, $\bar{x}$ ± *SD*) of their seasonal core ranges. We found support for our prediction that most vicuña seasonal core ranges overlap with those of other vicuñas–range sharing was high across measurement periods and sites in the park, whereby less than a fourth of the seasonal core ranges were exclusive (no portion shared with other vicuñas). Overlaps varied significantly across seasons (Friedman’s chi-squared = 8.08, d.f. = 3, *P* = 0.04)–however, the effect size was small (Kendall’s *W* = 0.245), and a post hoc Wilcoxon test with a Bonferroni correction resulted in no significant differences across pairs of seasons. In each seasonal analysis period, at least three and up to 10 vicuñas shared more than a third of their seasonal core ranges with other vicuñas.

### Diel migrations.—

**Fig. 2.**
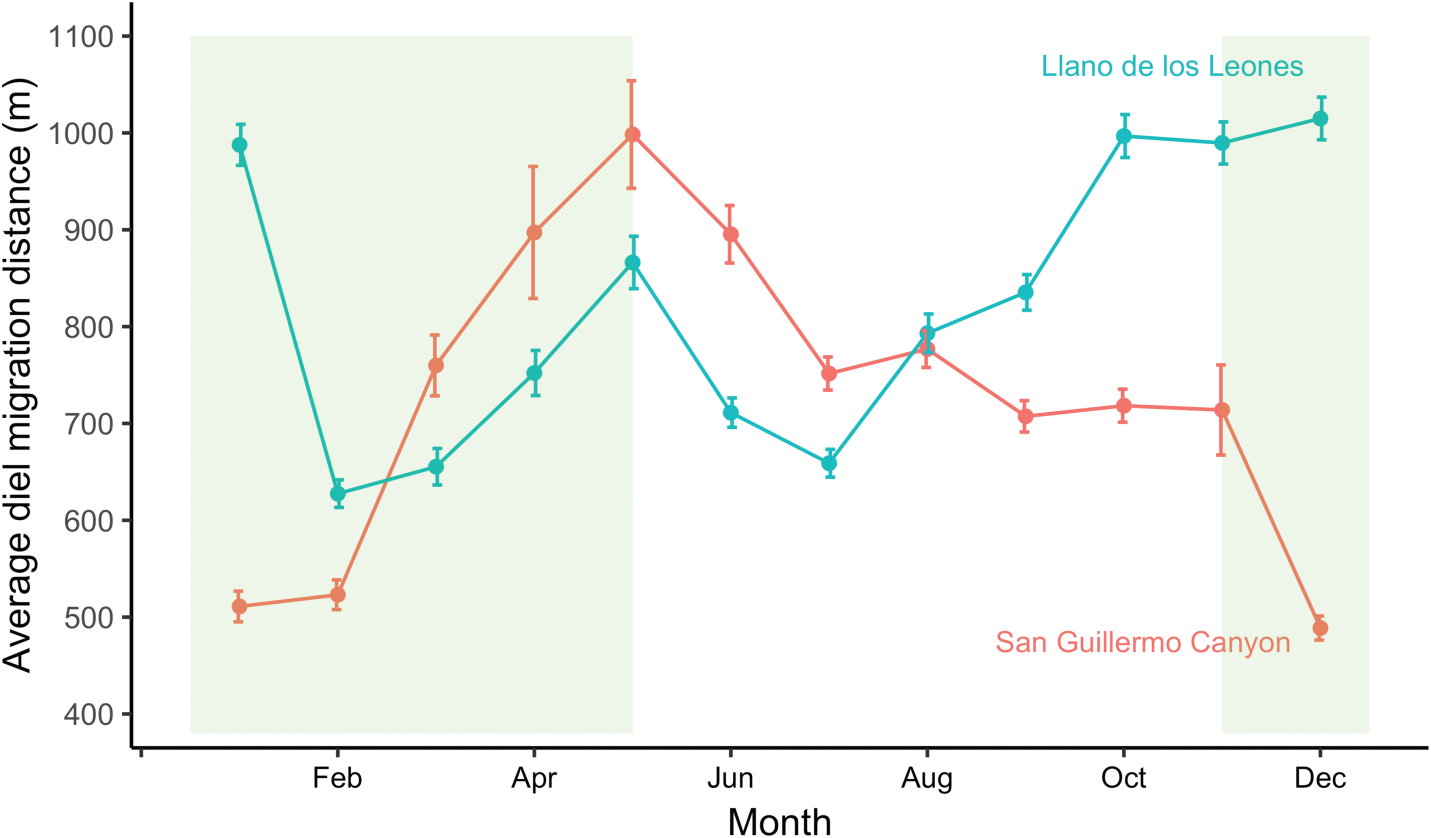
—A comparison of diel migration distances calculated on a monthly basis for vicuñas in the Llano de los Leones and San Guillermo Canyon for the duration of the study. Diel migration distances increased during the nongrowing period in the Llano de los Leones as compared to the San Guillermo Canyon where distances peak at the beginning of the nongrowing period and subsequently decline. The bars represent 95% confidence intervals based on standard errors. The shaded area represents the growing season.

Vicuña daily movement between day and night areas differed between the two sites and across seasons. Vicuñas in Llano de los Leones moved 822.4 m (95% confidence interval [*CI*] 810.9–833.9) on average during diel migrations between their day and night core areas, significantly more (*W* = 62,007,750, *P* < 0.001) than the 724.8 m (95% *CI* 708.9–740.6) average daily movement in the San Guillermo Canyon. Average daily distance moved between day and night areas also differed significantly (*t* = −3.35, *P* = 0.001) between the growing and nongrowing seasons, whereby the distance between day and night areas was on average 763.4 m (95% *CI* 748.3–778.5) in the growing season compared to 796.4 m (95% *CI* 784.3–808.4) in the nongrowing season. Vicuña individuals moved a total of 291.9 km (95% *CI* 260.9–322.9) annually, with a range of 145.5–440.3 km. Diel migration distances increased during the nongrowing period in the Llano de los Leones, peaking early in the growing period, as compared to the San Guillermo Canyon, where distances peak at the beginning of the nongrowing period and subsequently decline (Fig. 2). Night ranges were not clustered together with other vicuñas and differed in their relationship to seasonal core ranges between sites–vicuñas in the Llano de los Leones used distinct areas in the more open uplands (areas that represented lower predation risk), while those in the San Guillermo Canyon used higher-elevation areas of their day seasonal core ranges.

## Discussion

We studied vicuña spatial and social ecology using GPS location data in the southern end of its range in the Andes. Although some understanding of vicuña space use exists (Koford 1957; Franklin 1974, 1976; Arzamendia et al. 2018), previous studies are based on visual observations of marked animals, as opposed to GPS location data sets that allow for the ability to investigate animal space use and movement continuously across large temporal and spatial scales. Past research was also generally conducted in areas where vicuñas share landscapes with people to some degree, and in areas with relatively higher levels of precipitation (Koford 1957; Franklin 1976; Arzamendia et al. 2018). By contrast, the extremely remote location of our study site affords us a baseline picture of a species otherwise exposed to significant disturbance and threats elsewhere in its range. Additionally, the considerably lower levels of precipitation at our study site (Salvioli 2007) may affect space use and space sharing, offering an opportunity to gain insights on how space use in the species changes across environmental gradients.

Vicuñas in San Guillermo National Park were sedentary (i.e., did not undertake seasonal migrations) and demonstrated high site fidelity, in line with previous studies that observed year-round maintenance of territories by vicuña families (Franklin 1974, 1976; Bosch and Svendsen 1987). Collared vicuñas did not move between Llano de los Leones and San Guillermo Canyon, the two sites examined within the park. The home range estimates from our study were more than twice as large as previously reported (Table 1; Koford 1957; Franklin 1976; Arzamendia et al. 2018). We contend that the differences in range sizes could be a result of other studies being conducted in areas with higher precipitation levels and therefore higher primary productivity. For instance, the Pozuelos Biosphere Reserve in Argentina receives 46% more annual precipitation than San Guillermo National Park (Arzamendia et al. 2018). Koford (1957) suggested that vicuñas may use much larger territories, up to 1.01 km^2^, in barren parts of their range. Our results are also in line with space use predictions under the resource dispersion hypothesis, which suggests that clumped resources are likely to increase territory size (Macdonald 1983; Johnson et al. 2002). While this is a possibility, the differences in home range size could also result from methodological differences, since estimates from previous studies were based on a visual estimation of movements in the landscape rather than using quantitative home range estimation methods based on systematic data collection over sustained periods of time, as is possible with GPS collar data. Studies based on data obtained through visual observations have limitations in terms of obtaining a sufficient number of locations for determining accurate home ranges, with the data likely not meeting asymptotic requirements (Laver and Kelly 2008).

Vicuña seasonal core ranges were similar in size across the two sites in the park, despite the differences in NDVI, elevation, and slope between the Llano de los Leones and the San Guillermo Canyon, and the fact that NDVI acts as a spatial anchor for vicuñas (very limited habitat where vicuñas are drawn to due to high forage availability; Smith et al. 2019b). One exception occurred in the nongrowing period in 2014, when seasonal core ranges in the San Guillermo Canyon were more than twice as large as those in the Llano de los Leones, a result at odds with recent research that offers strong support for the habitat productivity hypothesis (Seigle-Ferrand et al. 2021). However, given the fact that neither seasonal home range nor seasonal core range sizes significantly change across analysis periods, we ascribe the difference in the core ranges to the inherent stochasticity associated with space use. Despite differences in plant phenology in the study area across the growing and nongrowing periods, seasonal core range sizes did not change across these periods, contrary to observations from previous studies that reported seasonal changes in territory size (Koford 1957). The absence of seasonal variation in seasonal core range size aligns with the very weak relationships observed between seasonal core range size and mean NDVI and seasonal core range size and family group size, also at odds with previously reported observations for the species (Franklin 1976; Arzamendia et al. 2018).

In contrast with previous studies, we found considerable evidence for tolerance of conspecifics from different families, especially while foraging (Franklin 1974, 1976)–most vicuña seasonal core ranges in San Guillermo National Park overlapped with seasonal core ranges of other individuals, with very few exclusive core ranges. This was corroborated by visual observations recorded opportunistically during the study period that revealed as many as five collared vicuñas from distinct family groups feeding in close proximity to each other on multiple occasions. Except for a few individuals, space sharing varied across seasons, seemingly in a stochastic manner–we did not detect systematic differences in seasonal core range overlap between the four seasons, despite earlier studies reporting increased territorial behavior during the breeding and birthing periods (Franklin 1976). Instead, we observed significant continuity of seasonal core ranges within individuals and considerable variation in proportions of seasonal core ranges shared with other sampled individuals (i.e., range overlap), which may suggest personality differences in terms of varying levels of aggression and repulsion behavior displayed by males in the same population (Franklin 1974). We conclude that vicuñas in the park deviate from the behavior of strictly exclusive-use territories described in some previous studies on this species (Koford 1957; Franklin 1974; Arzamendia et al. 2018; but see Vilá and Roig 1992; Vilá 1994; Cassini et al. 2009) in line with the predictions of the resource dispersion hypothesis (Macdonald 1983; Johnson et al. 2002). The results reported by Arzamendia et al. (2018) are especially comparable with our study, given the use of a quantitative method for home range estimation and that both studies were conducted on the southern vicuña subspecies, *L. v. vicugna*. At the same time, we acknowledge the limitations of comparing our results with those from Koford (1957) and Franklin (1974), given that territoriality and territories are largely behavioral concepts that are difficult to test based on purely remotely obtained data, as opposed to home ranges that can be reasonably derived from biologging animal location data.

Vicuña families in the park did not maintain clustered communal night ranges, instead largely choosing to use smaller areas within their seasonal home ranges or other areas in the open uplands. While vicuñas in both sites moved to higher-elevation areas for the night (Supplementary Data SD3), the flatter, less rugged terrain in the Llano de los Leones possibly results in vicuñas moving longer distances to reach their preferred night areas. Another possible factor may be that vicuñas in the Llano de los Leones may prefer to move farther away from the day foraging sites due to the high predation risk associated with the day sites (Smith et al. 2019a). However, given that vicuñas do not use some of the even-higher areas within the park, it is likely that elevation is one of several factors that vicuñas use to select refuge sites.

Although vicuñas in San Guillermo National Park did not migrate on a seasonal basis and maintained year-round home ranges, they undertook diel migrations, moving from the highly productive high-quality forage areas often located at relatively lower altitudes to the low productive open plains located at high altitudes. With the open plains in the park representing safe areas with low predation risk (Smith 2019a), vicuñas use diel migrations as an antipredator strategy in San Guillermo National Park (Smith 2019a). Diel migrations have been well-studied in marine environments (Neilson and Perry 1990; Alonzo et al. 2003; Hays 2003); however, their understanding in terrestrial systems is limited to a few species, such as plains zebras (*Equus quagga*; Courbin et al. 2019). Although vicuñas moved significantly longer average daily distances during the nongrowing period, the biological significance of this is likely to be limited due to the small difference across seasons. The differences in daily distances at the monthly level (Fig. 2), however, offer insights into the impacts of seasonal effects and differences in terrain at the two sites in the park. Vicuña daily distances reduced at the beginning of the growing season, likely due to the increased availability of forage, with reductions of 32% and 36%, respectively, in San Guillermo Canyon and Llano de los Leones. The decrease in daily distances in San Guillermo Canyon during the nongrowing season could be a strategy to save energy, when forage is limited and less nutritious. A more complex trade-off between managing predation risk, conserving energy, and achieving access to forage may explain the initial drop followed by a steady increase in daily distances in the nongrowing season in the Llano de los Leones, which comprises concentrated sources of vegetation in the low-lying areas, surrounded by plains. These daily movements, although not as dramatic or landscape-altering as long-distance seasonal migrations, may, however, be important due to the likely significant energetic costs involved. With increasing options for including energetics assessments in GPS collars, we propose that future work on the species assesses the energetic impacts of these daily migrations and compare them with long-distance seasonal migrations. Studies on vicuña populations in areas where large predators are functionally extinct and where the species does not face hunting pressures may also offer additional insights on this behavior.

Our analysis found several key differences with previous studies in vicuña behavior and space. Vicuña home ranges in San Guillermo were considerably larger than in previous estimates. Overlaps between home ranges were common, with most vicuñas sharing large portions of their home range with other individuals. Vicuña home range sizes did not undergo seasonal changes and did not vary with changing family sizes and availability of vegetation in the home range. Our work highlights the differences in behavior likely arising from a combination of environmental factors and the fact that the San Guillermo National Park vicuña populations are almost completely undisturbed by human activity. From a broader perspective, this study offers an insight into the plasticity of social behavior and tolerance of nonfamilial conspecifics in the species. In semiarid areas like San Guillermo National Park with plant growth occurring only during a short growing season, the limited availability of forage may preclude territorial behavior in feeding areas and increase tolerance of nonfamilial conspecifics during the day. Similar breakdowns in territorial behavior and increased tolerance of conspecifics when food availability is limited or concentrated in small geographical areas have been demonstrated in other mammalian (Newsome et al. 2013) and avian (Carpenter and MacMillen 1976) species. The flexibility in social behavior and space use displayed by the species may be instrumental in ensuring survival, and its recent resurgence, in this extremely arid and harsh landscape.

## Supplementary Data

Supplementary data are available at *Journal of Mammalogy* online.

**Supplementary Data SD1.**—Total number of GPS locations recorded with start and end dates and number of months with data for each vicuña in San Guillermo National Park.

**Supplementary Data SD2.**—Vicuña home range sizes in the two sites in San Guillermo National Park for different periods during the study: (a) overall core range sizes (km^2^) from April 2014 to February 2017, (b) seasonal home and seasonal core ranges in km^2^ for the 2014 nongrowing period, (c) seasonal home and seasonal core ranges in km^2^ for the 2014 growing period, (d) seasonal home and seasonal core ranges in km^2^ for the 2015 nongrowing period, and (e) seasonal home and seasonal core ranges in km^2^ for the 2015 growing period.

**Supplementary Data SD3.**—Centroids of day (triangles) and night (circles) locations of vicuñas in the (a) Llano de los Leones and (b) San Guillermo Canyon in San Guillermo National Park. Map scales differ for (a) and (b). All vicuñas except one in the San Guillermo Canyon moved to higher locations for the night.

gyad018_suppl_Supplementary_Data_S1

gyad018_suppl_Supplementary_Data_S2

gyad018_suppl_Supplementary_Data_S3
