# Supplementary material for: Spatial ecology of the Vicuña (Lama vicugna) in a high Andean protected area
Source: J Mammal. 2023 Mar 16;104(3):509–18. doi: 10.1093/jmammal/gyad018 (PMC10243966; doi:10.1093/jmammal/gyad018)
Supplement: gyad018_suppl_Supplementary_Data_S1 [file gyad018_suppl_supplementary_data_s1.docx]

| Vicuña ID | Total number of locations | | Start date | | End date | | | Number of months with data | |  |  |  |
| --- | --- | --- | --- | --- | --- | --- | --- | --- | --- | --- | --- | --- |
|  | | Llano de los Leones | | | | | | | | | |  |
| 14 | 946 | | | 03 May 2014 | | | 29 Aug 2014 | | 4 | |  |  |
| 16 | 7675 | | | 10 May 2014 | | | 24 Dec 2016 | | 32 | |  |  |
| 17 | 1127 | | | 15 May 2014 | | | 03 Oct 2014 | | 5 | |  |  |
| 18 | 4221 | | | 27 May 2015 | | | 05 Nov 2016 | | 17 | |  |  |
| 23 | 5832 | | | 01 Jun 2014 | | | 30 May 2016 | | 24 | |  |  |
| 24 | 6232 | | | 09 May 2014 | | | 25 Jun 2016 | | 25 | |  |  |
| 25 | 7741 | | | 07 May 2014 | | | 30 Dec 2016 | | 32 | |  |  |
| 27 | 1294 | | | 16 May 2014 | | | 25 Oct 2014 | | 5 | |  |  |
| 30 | 5690 | | | 07 May 2014 | | | 18 Apr 2016 | | 23 | |  |  |
| 33 | 1700 | | | 08 Jun 2015 | | | 07 Jan 2016 | | 7 | |  |  |
| 34 | 8085 | | | 12 May 2014 | | | 16 Feb 2017 | | 33 | |  |  |
| 35 | 5995 | | | 05 May 2014 | | | 24 May 2016 | | 25 | |  |  |
| 36 | 2884 | | | 16 May 2014 | | | 12 May 2015 | | 12 | |  |  |
|  | | San Guillermo Canyon | | | | | | | | | |  |
| 13 | 5259 | | | 08 Jun 2014 | | 27 Mar 2016 | | | 22 | | | |
| 15 | 1165 | | | 11 Jun 2015 | | 03 Nov 2015 | | | 5 | | | |
| 19 | 4381 | | | 01 May 2014 | | 31 Oct 2015 | | | 18 | | | |
| 20 | 6124 | | | 07 Jun 2014 | | 12 July 2016 | | | 25 | | | |
| 21 | 2565 | | | 17 May 2015 | | 01 April 2016 | | | 11 | | | |
| 22 | 5551 | | | 06 Jun 2014 | | 29 Apr 2016 | | | 23 | | | |
| 26 | 1257 | | | 07 June 2015 | | 11 Nov 2015 | | | 5 | | | |
| 28 | 1970 | | | 29 May 2015 | | 30 Jan 2016 | | | 8 | | | |
| 29 | 5595 | | | 10 May 2014 | | 08 Apr 2016 | | | 23 | | | |
| 31 | 1062 | | | 11 May 2015 | | 20 Sep 2015 | | | 4 | | | |
| 32 | 1521 | | | 30 April 2014 | | 06 Nov 2014 | | | 7 | | | |
