# Supplementary material for: Spatial ecology of the Vicuña (Lama vicugna) in a high Andean protected area
Source: J Mammal. 2023 Mar 16;104(3):509–18. doi: 10.1093/jmammal/gyad018 (PMC10243966; doi:10.1093/jmammal/gyad018)
Supplement: gyad018_suppl_Supplementary_Data_S2 [file gyad018_suppl_supplementary_data_s2.docx]

| Llano de los Leones | | San Guillermo Canyon | |
| --- | --- | --- | --- |
| Individual id | Overall core range (50%) | Individual id | Overall core range (50%) |
| 14 | 0.25 | 13 | 0.14 |
| 16 | 0.04 | 15 | 0.41 |
| 17 | 0.03 | 19 | 0.23 |
| 18 | 0.24 | 20 | 0.47 |
| 23 | 0.50 | 21 | 0.37 |
| 24 | 0.03 | 22 | 0.12 |
| 25 | 0.05 | 26 | 2.02 |
| 27 | 0.24 | 28 | 0.16 |
| 30 | 0.17 | 29 | 1.18 |
| 33 | 3.30 | 31 | 2.05 |
| 34 | 0.14 | 32 | 0.14 |
| 35 | 0.03 |  |  |
| 36 | 0.37 |  |  |

| Vicuña ID | Seasonal home range (95%) | | Seasonal core range (50%) | |
| --- | --- | --- | --- | --- |
|  | Day | Night | Day | Night |
| Llano de los Leones | | | | |
| 14 | 1.67 | 0.70 | 0.21 | 0.02 |
| 16 | 0.65 | 0.91 | 0.01 | 0.02 |
| 17 | 2.43 | 3.71 | 0.03 | 0.15 |
| 23 | 0.39 | 0.40 | 0.01 | 0.03 |
| 24 | 0.32 | 0.73 | 0.02 | 0.01 |
| 25 | 1.54 | 1.34 | 0.01 | 0.02 |
| 27 | 3.84 | 2.85 | 0.23 | 0.36 |
| 30 | 4.57 | 5.33 | 0.39 | 0.51 |
| 34 | 2.03 | 1.03 | 0.02 | 0.04 |
| 35 | 0.44 | 1.15 | 0.01 | 0.01 |
| 36 | 7.11 | 3.98 | 0.29 | 0.30 |
| San Guillermo Canyon | | | | |
| 13 | 2.51 | 0.42 | 0.15 | 0.02 |
| 19 | 1.92 | 1.13 | 0.29 | 0.07 |
| 20 | 1.72 | 0.71 | 0.23 | 0.04 |
| 22 | 3.41 | 0.46 | 0.36 | 0.02 |
| 29 | 6.33 | 1.81 | 0.35 | 0.05 |
| 32 | 5.24 | 4.20 | 0.14 | 0.14 |

| Vicuña ID | Seasonal home range (95%) | | Seasonal core range (50%) | |
| --- | --- | --- | --- | --- |
|  | Day | Night | Day | Night |
| Llano de los Leones | | | | |
| 16 | 1.52 | 0.54 | 0.06 | 0.01 |
| 23 | 13.74 | 3.93 | 0.41 | 0.09 |
| 24 | 0.29 | 0.22 | 0.03 | 0.01 |
| 25 | 5.19 | 2.96 | 0.10 | 0.33 |
| 30 | 7.41 | 2.07 | 0.02 | 0.21 |
| 34 | 7.54 | 3.38 | 0.33 | 0.82 |
| 35 | 1.94 | 1.73 | 0.01 | 0.12 |
| 36 | 13.91 | 7.14 | 0.36 | 0.38 |
| San Guillermo Canyon | | | | |
| 13 | 2.89 | 0.29 | 0.11 | 0.02 |
| 19 | 3.95 | 1.19 | 0.11 | 0.05 |
| 20 | 3.64 | 1.49 | 0.33 | 0.10 |
| 22 | 1.61 | 0.27 | 0.07 | 0.02 |
| 29 | 34.79 | 18.15 | 0.33 | 0.07 |

| Vicuña ID | Seasonal home range (95%) | | Seasonal core range (50%) | |
| --- | --- | --- | --- | --- |
|  | Day | Night | Day | Night |
| Llano de los Leones | | | | |
| 16 | 0.88 | 0.61 | 0.02 | 0.01 |
| 18 | 9.07 | 5.86 | 0.22 | 0.10 |
| 23 | 22.63 | 18.36 | 2.37 | 0.12 |
| 24 | 0.82 | 0.64 | 0.02 | 0.01 |
| 25 | 1.65 | 1.71 | 0.01 | 0.21 |
| 30 | 3.81 | 3.22 | 0.01 | 0.20 |
| 33 | 18.95 | 8.47 | 2.28 | 0.88 |
| 34 | 3.17 | 2.71 | 0.01 | 0.47 |
| 35 | 4.74 | 1.69 | 0.04 | 0.12 |
| San Guillermo Canyon | | | | |
| 13 | 7.64 | 2.57 | 0.23 | 0.02 |
| 15 | 3.64 | 1.09 | 0.38 | 0.05 |
| 19 | 5.16 | 3.08 | 0.25 | 0.09 |
| 20 | 2.83 | 2.35 | 0.52 | 0.19 |
| 21 | 9.28 | 2.63 | 0.56 | 0.12 |
| 22 | 3.30 | 1.16 | 0.19 | 0.01 |
| 26 | 12.28 | 3.07 | 2.02 | 0.50 |
| 28 | 3.43 | 2.49 | 0.36 | 0.25 |
| 29 | 37.57 | 16.16 | 1.64 | 0.41 |
| 31 | 14.46 | 3.59 | 3.08 | 1.07 |

| Vicuña ID | Seasonal home range (95%) | | Seasonal core range (50%) | |
| --- | --- | --- | --- | --- |
|  | Day | Night | Day | Night |
| Llano de los Leones | | | | |
| 16 | 0.89 | 0.20 | 0.02 | 0.01 |
| 18 | 2.78 | 2.23 | 0.18 | 0.12 |
| 23 | 12.20 | 2.63 | 1.02 | 0.14 |
| 24 | 0.57 | 0.31 | 0.06 | 0.02 |
| 25 | 4.64 | 4.12 | 0.12 | 0.11 |
| 30 | 5.44 | 5.82 | 0.36 | 0.49 |
| 34 | 2.58 | 1.55 | 0.07 | 0.05 |
| 35 | 1.40 | 1.54 | 0.01 | 0.04 |
| San Guillermo Canyon | | | | |
| 13 | 1.68 | 0.57 | 0.06 | 0.02 |
| 20 | 6.77 | 1.91 | 0.41 | 0.38 |
| 21 | 1.14 | 0.45 | 0.19 | 0.03 |
| 22 | 1.76 | 0.25 | 0.09 | 0.02 |
| 29 | 2.58 | 0.71 | 0.47 | 0.13 |
