# Supplementary figures and images for: Spatial ecology of the Vicuña (Lama vicugna) in a high Andean protected area
Source: J Mammal. 2023 Mar 16;104(3):509–18. doi: 10.1093/jmammal/gyad018 (PMC10243966; doi:10.1093/jmammal/gyad018)

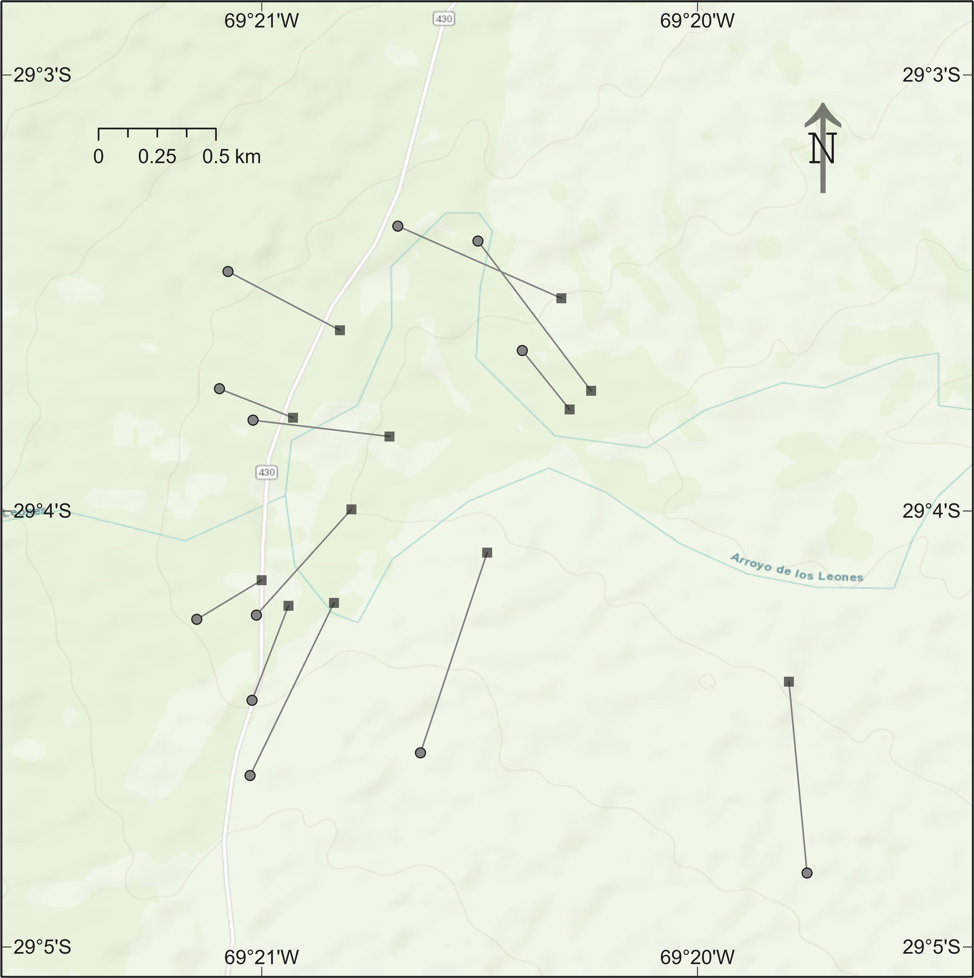


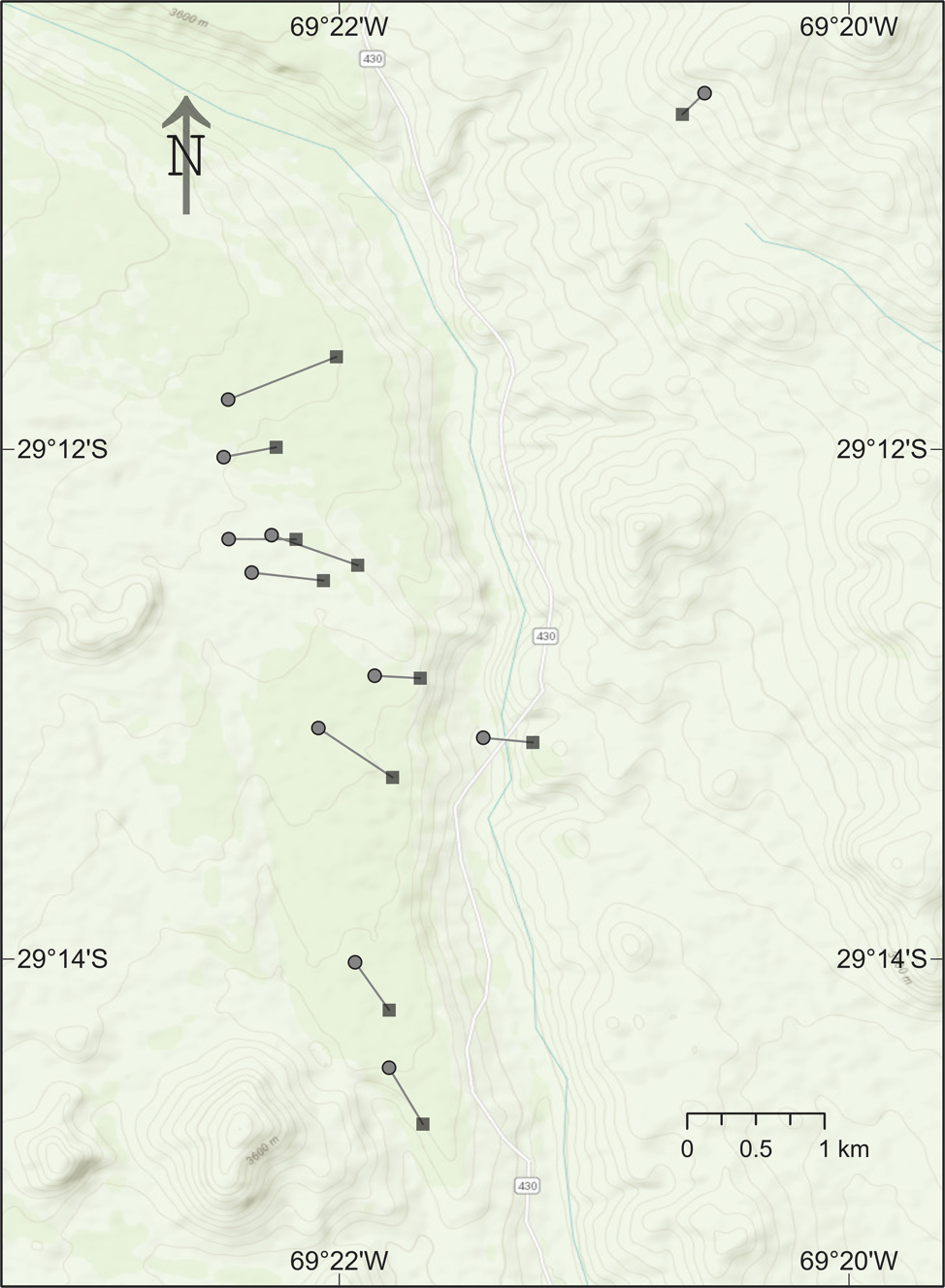

Supplement: gyad018_suppl_Supplementary_Data_S3 [file gyad018_suppl_supplementary_data_s3.docx]
